# A quantitative evaluation of aerosol generation during cardiopulmonary resuscitation

**DOI:** 10.1111/anae.16162

**Published:** 2023-11-03

**Authors:** A. J. Shrimpton, V. Brown, J. Vassallo, J. P. Nolan, J. Soar, F. Hamilton, T. M. Cook, B. R. Bzdek, J. P. Reid, C. H. Makepeace, J. Deutsch, R. Ascione, J. M. Brown, J. R. Benger, A. E. Pickering

**Affiliations:** ^1^ Anaesthesia, Pain and Critical Care Sciences, School of Physiology, Pharmacology and Neuroscience University of Bristol Bristol UK; ^2^ Critical Care, South Western Ambulance Service NHS Foundation Trust UK; ^3^ Great Western Air Ambulance Charity Bristol UK; ^4^ Institute of Naval Medicine Gosport UK; ^5^ Academic Department of Military Emergency Medicine Royal Centre for Defence Medicine Birmingham UK; ^6^ University of Warwick, Warwick Medical School Coventry UK; ^7^ Department of Anaesthesia and Intensive Care Medicine Royal United Hospital Bath UK; ^8^ Department of Anaesthesia and Intensive Care Medicine North Bristol NHS Trust Bristol UK; ^9^ MRC Integrative Epidemiology Unit University of Bristol UK; ^10^ Department of Anaesthesia and Intensive Care Medicine Royal United Hospital Bath UK; ^11^ School of Chemistry University of Bristol Bristol UK; ^12^ School of Chemistry University of Bristol Bristol UK; ^13^ Langford Vets and Translational Biomedical Research Centre University of Bristol UK; ^14^ Langford Vets and Translational Biomedical Research Centre University of Bristol UK; ^15^ Translational Biomedical Research Centre University of Bristol Bristol UK; ^16^ University Hospital Bristol Weston NHS Trust Bristol UK; ^17^ Faculty of Health and Applied Sciences University of the West of England Bristol UK; ^18^ Department of Anaesthesia University Hospitals Bristol and Weston Bristol UK; ^19^ Anaesthesia, Pain and Critical Care Sciences, School of Physiology, Pharmacology and Neuroscience University of Bristol Bristol UK

**Keywords:** aerosol‐generating procedure, cardiopulmonary resuscitation, CPR, out‐of‐hospital cardiac arrest

## Abstract

It is unclear if cardiopulmonary resuscitation is an aerosol‐generating procedure and whether this poses a risk of airborne disease transmission to healthcare workers and bystanders. Use of airborne transmission precautions during cardiopulmonary resuscitation may confer rescuer protection but risks patient harm due to delays in commencing treatment. To quantify the risk of respiratory aerosol generation during cardiopulmonary resuscitation in humans, we conducted an aerosol monitoring study during out‐of‐hospital cardiac arrests. Exhaled aerosol was recorded using an optical particle sizer spectrometer connected to the breathing system. Aerosol produced during resuscitation was compared with that produced by control participants under general anaesthesia ventilated with an equivalent respiratory pattern to cardiopulmonary resuscitation. A porcine cardiac arrest model was used to determine the independent contributions of ventilatory breaths, chest compressions and external cardiac defibrillation to aerosol generation. Time‐series analysis of participants with cardiac arrest (n = 18) demonstrated a repeating waveform of respiratory aerosol that mapped to specific components of resuscitation. Very high peak aerosol concentrations were generated during ventilation of participants with cardiac arrest with median (IQR [range]) 17,926 (5546–59,209 [1523–242,648]) particles.l^‐1^, which were 24‐fold greater than in control participants under general anaesthesia (744 (309–2106 [23–9099]) particles.l^‐1^, p < 0.001, n = 16). A substantial rise in aerosol also occurred with cardiac defibrillation and chest compressions. In a complimentary porcine model of cardiac arrest, aerosol recordings showed a strikingly similar profile to the human data. Time‐averaged aerosol concentrations during ventilation were approximately 270‐fold higher than before cardiac arrest (19,410 (2307–41,017 [104–136,025]) vs. 72 (41–136 [23–268]) particles.l^‐1^, p = 0.008). The porcine model also confirmed that both defibrillation and chest compressions generate high concentrations of aerosol independent of, but synergistic with, ventilation. In conclusion, multiple components of cardiopulmonary resuscitation generate high concentrations of respiratory aerosol. We recommend that airborne transmission precautions are warranted in the setting of high‐risk pathogens, until the airway is secured with an airway device and breathing system with a filter.

## Introduction

Whether cardiopulmonary resuscitation (CPR) generates high concentrations of respiratory aerosol is uncertain and controversial [1, 2, 3]. Most resuscitation guidelines have adopted a precautionary approach, prioritising rescuer safety and designating CPR as an aerosol‐generating procedure [2, 4, 5, 6]. These precautions, designed to reduce the risk of airborne disease transmission, may delay initiation of resuscitation with consequent impact on survival whilst rescuers don personal protective equipment [4]. This approach may have contributed to worse outcomes following cardiac arrest during the COVID‐19 pandemic [7, 8]. It remains unknown whether the precautionary approach confers appropriate protection to providers or causes unnecessary and inappropriate delays to commencement of CPR.

Case reports and retrospective epidemiological studies suggest an association between performing CPR and the risk of airborne disease transmission [9, 10, 11, 12]. However, to our knowledge, the amount of respiratory aerosol generated during CPR has never been measured in humans. This reflects the inherent challenges of using highly sensitive aerosol detection devices in a range of uncontrolled environments given the unpredictable nature of cardiac arrest.

The risk of making assumptions about aerosol generation from medical procedures is well illustrated by recent clinical quantitative aerosol studies. These studies show that several procedures previously considered to be high risk for aerosol generation, such as tracheal intubation and facemask ventilation, do not generate high concentrations of aerosol [13, 14, 15, 16, 17, 18, 19]. This work has resulted in their removal from the list of aerosol‐generating procedures in England [20, 21]. It is important to ensure appropriate precautions are being taken by rescuers performing CPR whilst minimising the risk of patient harm. As such, there is a pressing need to quantify respiratory aerosol generation during CPR in humans to help inform infection prevention and control guidelines.

We conducted a prospective study to quantify aerosol generation in patients with out‐of‐hospital cardiac arrest to test the hypothesis that CPR generates high concentrations of respiratory aerosol. As we were unable to measure respiratory aerosol concentrations in participants before cardiac arrest, or to separate individual components of CPR during cardiac arrest management, we conducted complementary investigations in human participants under elective general anaesthesia without cardiac arrest, and in a porcine cardiac arrest model, to measure the extent and nature of aerosol generation during each CPR component.

## Methods

Ethics approval for the human studies was granted by the Greater Manchester Research Ethics Committee. Human participants with cardiac arrest were recruited prospectively via deferred nominated consultee consent following an initial consent waiver. The patients who participated in the general anaesthesia part of the study gave written consent before enrolment. The pigs were made available to the study team from two separate research projects (project licences PC545FB99 and PP4585512, UK Home Office). Approval to study aerosol generation in these animals was granted by the University of Bristol Animal Welfare and Ethical Review Body in accordance with the 3Rs animal welfare principles (UIN‐22‐087) [22].

The primary outcome measure was to determine the respiratory aerosol concentrations generated by ventilation during CPR (compared with that generated in control participants under general anaesthesia). Secondary outcome measures included determination of aerosol generation by chest compressions and defibrillation. This is the first study of its kind and so no relevant data were available to inform a power calculation. The planned number of human subjects (n = 18) was estimated from our previous aerosol sampling studies, which have been adequately powered to detect aerosol generated from natural respiratory events or aerosol‐generating procedures [14, 15, 16, 23, 24]. A power calculation was conducted for the subsequent porcine study, based on a two‐tailed, non‐parametric paired comparison of aerosol generation before and following cardiac arrest. The human data comparing ventilation of participants under general anaesthesia vs. ventilation of participants with cardiac arrest resulted in an estimated effect size of 1.67. Therefore, eight pigs were required to achieve a power of 95% (α 0.05) [25].

For patients in cardiopulmonary arrest, aerosol recordings were non‐invasive and inclusion in the study did not alter standard clinical care. The first‐to‐scene paramedics provided standardised advanced life support to adults aged ≥ 18 y in cardiac arrest, comprising insertion of a supraglottic airway device (i‐gel®, Intersurgical, Wokingham, UK) and CPR according to Resuscitation Council UK guidelines (a ratio of 30:2 compressions to breaths) [5, 26]. Patients were supine and received either manual or mechanical chest compressions (Lund University Cardiac Assist System (LUCAS), Stryker, Sweden).

Aerosol recording started after arrival of a designated pre‐hospital critical care team with an additional researcher. The researcher timestamped all events including: ventilatory breaths; chest compressions; defibrillation; rhythm checks; breathing system disconnections; and return of spontaneous circulation. Recordings were terminated if the i‐gel was exchanged for a tracheal tube, or the airway was contaminated by gastric contents.

**Figure 1:**
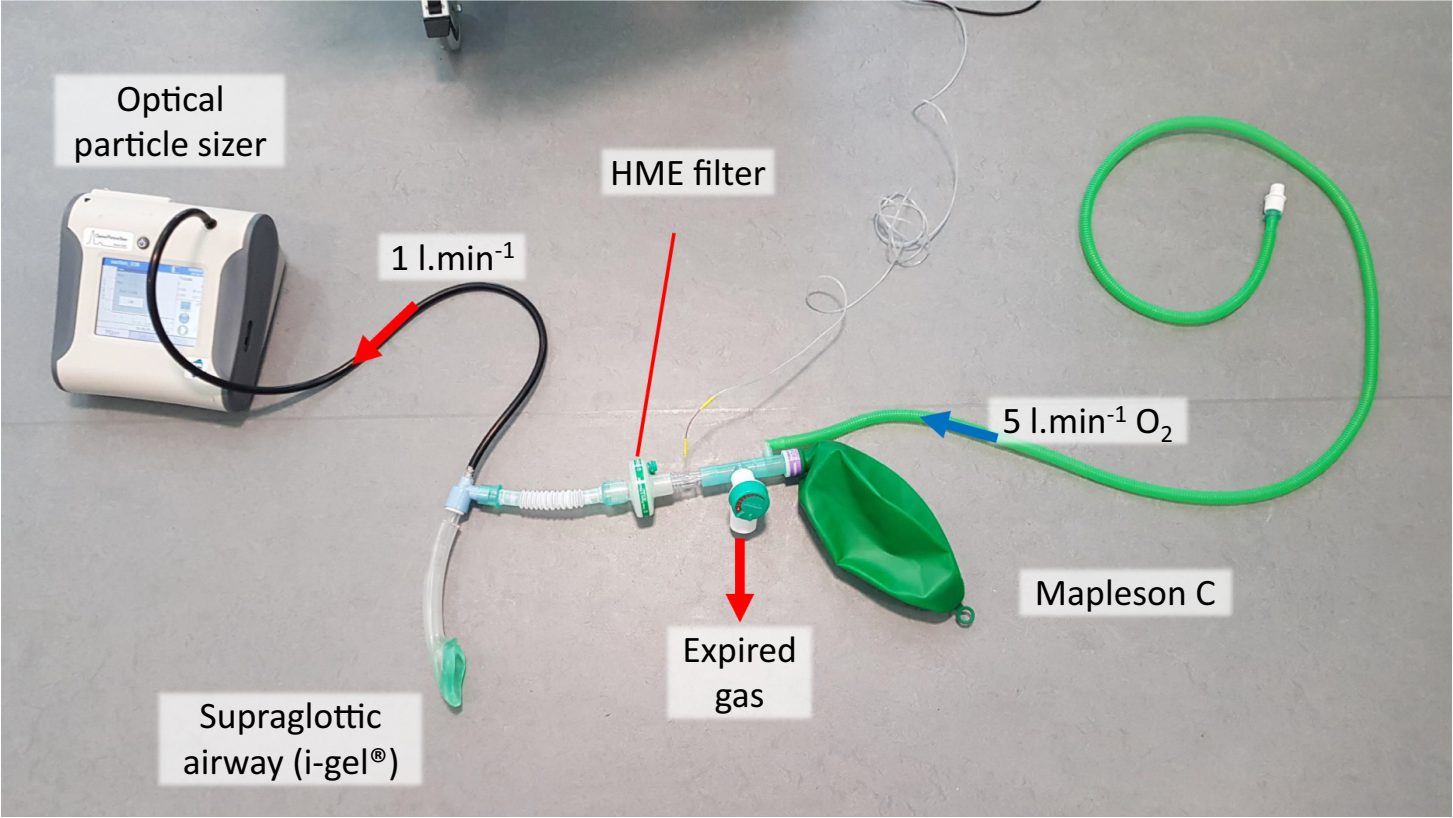
Breathing system with aerosol sampling used for all studies. HME, heat and moisture exchange filter.

Aerosol samples were collected from a closed breathing system (Fig. 1). Patients' lungs were manually ventilated using a Mapleson C breathing system connected to the i‐gel, with an oxygen flow of 5 l.min^‐1^. An optical particle sizer (Model 3330, TSI Incorporated, Shoreview, MN, USA) sampled gas from the circuit at a flow rate of 1 l.min^‐1^ and detected aerosol in the respirable size range (0.3–10 μm diameter, at 1 Hz). An inline filter (placed distal to the point of aerosol sampling) removed 99.99% of particles > 0.3 μm in diameter to ensure sampled particles originated from the patient and not the environment or supplementary oxygen. The defibrillator (X‐series, Zoll, Asahi Kasei, Chelmsford, MA, USA) recorded capnography for all patients; end‐tidal carbon dioxide (ETCO_2_) data were obtained from 9/18 patients.

Sixteen patients who required anaesthesia for elective surgery were recruited as a non‐cardiac arrest, ventilated comparator group. Patients were aged > 18 y; ASA physical status ≤ 3; and were planned to have general anaesthesia with a supraglottic airway device. Patients were anaesthetised using total intravenous anaesthesia with propofol and underwent a period of manual ventilation using the same sampling setup as the CPR study (Fig. 1). A ventilatory pattern of two breaths followed by a 16 s pause was used immediately after insertion of the i‐gel® (whilst the participant was apnoeic). This procedure was designed to replicate the ventilatory pattern delivered during CPR with a 30:2 compression‐to‐ventilation ratio but without chest compressions. The ETCO_2_ data were retrieved from the anaesthetic machine (Aisys^2^, GE Datex‐Ohmeda, Madison, WI, USA) using ARCTool software (GE Healthcare, Chicago, IL, USA).

Following collection of the observational human data, it became clear that we were unable to isolate the contribution of individual components of CPR to aerosol generation. To address this, we developed a porcine model of cardiac arrest. This was used to simulate bystander CPR; compare aerosol generation from ventilatory breaths during CPR with those before cardiac arrest; and explore the independent and combined contributions of external cardiac defibrillation and chest compressions on aerosol generation. The porcine work was conducted in a high‐specification hybrid operating theatre within the Translation Biomedical Research Centre at the University of Bristol. Aerosol was sampled from within the breathing system via the tracheal tube using the same sampling technique as for the human studies (Fig. 1). This approach enabled respiratory aerosol identification by excluding the influence of environmental/background aerosol particles and room ventilation. Data were collected from pigs involved in two pre‐existing research projects; CPR was undertaken following euthanasia after the studies had reached their pre‐defined study endpoints. Sixty seconds of sampling were recorded during manual ventilation of the anaesthetised animals before cardiac arrest, to act as a baseline comparator. In seven pigs, the sampling circuit was disconnected, and euthanasia performed with pentobarbital sodium (140 mg.kg^‐1^, intravenously). The remaining pig sustained a cardiac arrest under anaesthesia and was unable to be resuscitated. Following confirmation of death, a rapid bolus of potassium chloride (50 mmol, intravenously) was administered to all animals to ensure rapid onset of cardiac arrhythmia (VF or asystole). The aerosol sampling circuit was reattached to the tracheal tube and aerosol was sampled continuously during the pre‐defined CPR protocol (online Supporting Information Appendix S1). One animal developed severe pulmonary oedema during the final phase of 30:2 chest compressions at which point the recording was discontinued. Pigs were supine and chest compressions were delivered manually. Shocks were either 100 J (LifePak 20, Medtronic, Watford, UK) or 120 J (X‐series, Zoll, Asahi Kasei, Chelmsford, MA, USA). The Zoll device also recorded ETCO_2_, spirometry and the electrocardiogram rhythm strip.

Aerosol data were processed using Aerosol Instrument Manager software (v10.3, TSI incorporated, Shoreview, MN, USA) and analysed using R studio (v4.2.2, R Foundation, Vienna, Austria). Peak and average values were calculated over specific time windows corresponding to breaths, chest compressions or defibrillator shocks. The peak concentration is the highest value recorded during the event (most relevant for events with brief spikes in aerosol, such as defibrillation or individual breaths), the ‘time‐average’ concentration is the total aerosol concentration of the event, divided by its duration (which is more appropriate to compare prolonged periods of exposure such as chest compressions). Particle size distribution analysis was undertaken using normalised aerosol concentrations (dN/dlogD_p_) to account for the sampling bin widths and lognormal particle size distribution; dN is the total concentration of particles and dlogD_p_ is the difference in the log of the bin width [27]. Aerosol concentrations recorded per cm^3^ were converted to litres to enhance clarity. Non‐parametric analyses (Wilcoxon) were used to compare aerosol data which were not normally distributed (Shapiro‐Wilk). Results are reported as median (IQR [range]) unless otherwise stated.

The two breaths, delivered as pairs in the 30:2 CPR sequences, produced different waveforms of aerosol because expired gas from the first breath (breath 1) was purged by delivery of fresh gas from the second breath (breath 2). Breath 1 therefore comprised a single ventilatory breath in all study subjects, enabling comparison between groups. In contrast, breath 2 contained a ventilatory breath plus a period of external chest compressions when delivered during CPR in humans and pigs (but not in the control participants under general anaesthesia). As these two breaths were not equivalent events, ventilation‐associated aerosol concentration analysis was undertaken separately during two periods: breath 1, the 4 s after the first ventilatory breath (identified as the nadir before the first peak); and breath 2, the subsequent 12 s. Periods were excluded from the ventilation analysis if there was a rhythm check, defibrillation shock or other activity adjacent to the ventilatory breath to ensure these components had no impact on aerosol concentrations detected.

## Results

Complete recordings were obtained during resuscitation of 18 adults in out‐of‐hospital cardiac arrest (11 male, median (IQR [range]) age 59 (56–75 [40–85]) y). All patients had bystander CPR before arrival of the paramedics. The initial cardiac rhythm was asystole in 13 patients, pulseless electrical activity in three patients, and ventricular fibrillation in two patients. Four patients received external cardiac defibrillation during aerosol sampling. Median time between the initial call to emergency services and start of aerosol sampling was 32 (24–43 [10–55]) min. One patient was successfully resuscitated and transferred to hospital, the other 17 were declared dead at the scene. There was no deviation from standard resuscitation care and no adverse events were associated with aerosol sampling for any patient.

**Figure 2:**
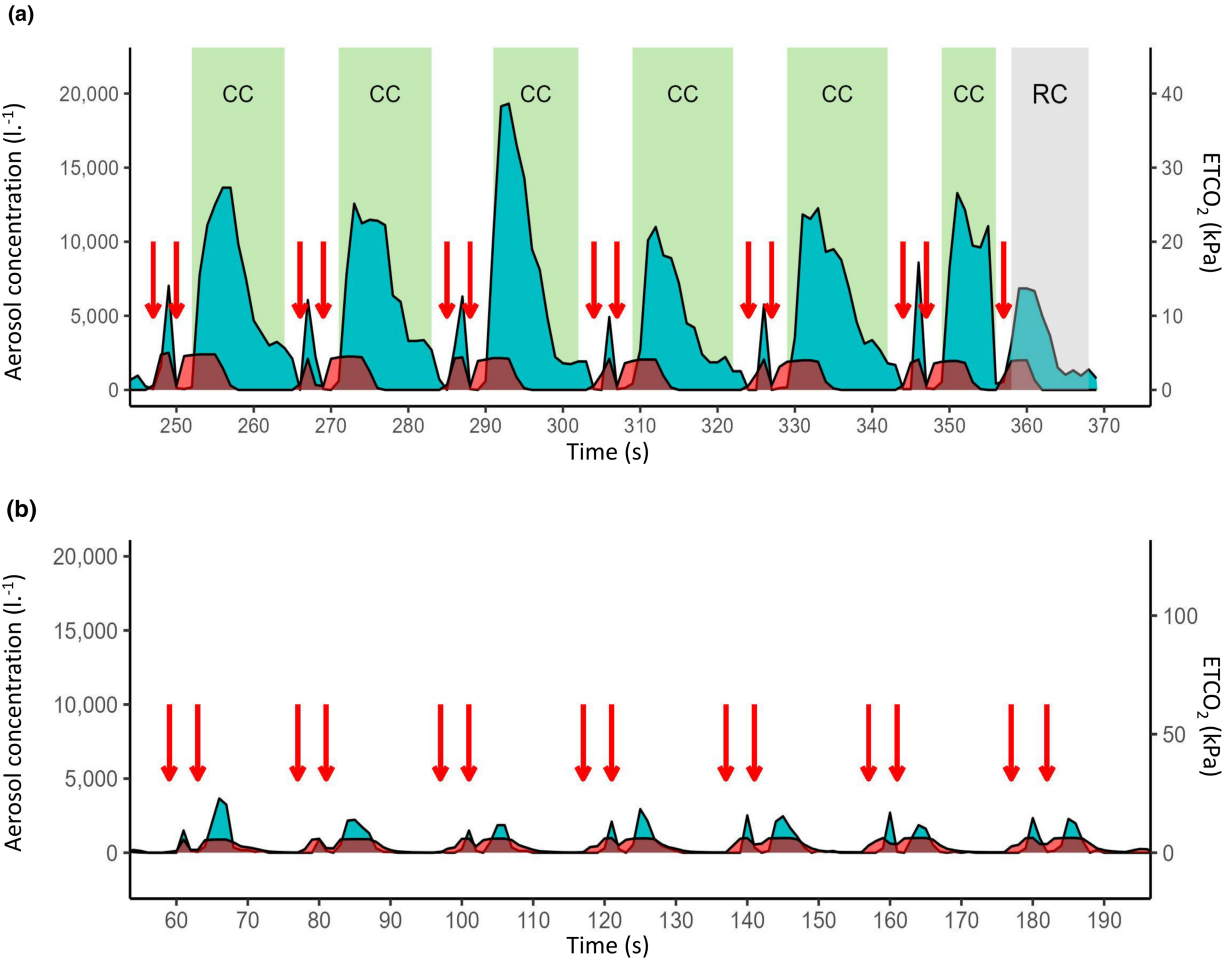
Comparison of timelines demonstrating aerosol concentrations and their association with exhaled carbon dioxide recorded during sampling. Blue, aerosol concentration; red, ETCO_2_; red arrows, ventilatory breaths. Note same y‐axis for comparison. (a) Representative timeline for a participant with cardiac arrest during CPR (30:2 compressions to breaths). This shows the repeating pattern of aerosol peaks associated with ventilatory breaths (seen also on corresponding ETCO_2_ trace). Note the prolonged aerosol plateau associated with the second breath and chest compressions; (b) aerosol concentrations generated by an equivalent pattern of ventilation in a participant under general anaesthesia and not in cardiac arrest, receiving two ventilatory breaths and no chest compressions. CC, chest compressions; RC, rhythm check.

Median (IQR [range]) duration of aerosol sampling was 7.3 (5.3–10.7 [2.0–16.1]) min. This showed a consistent, repeating pattern of aerosol generation that matched the timestamped sequence of breaths and chest compressions periods (Fig. 2a). This was corroborated when the ETCO_2_ waveforms were superimposed, showing a very similar pattern confirming the association of aerosol concentration peaks with the expiratory phase of ventilation.

During 30:2 compression to ventilation CPR, the aerosol profile of the first breath of each pair (breath one: comprising a single breath) showed a much sharper peak compared with the prolonged, slowly decaying plateau that lasted > 10 s seen with the second breath (breath two: composed of a breath plus chest compressions). Time‐averaged aerosol concentrations were greater for breath 2 compared with breath one (p < 0.001, Table 1), and the breath two aerosol wave consistently outlasted the decay in the ETCO_2_ trace sampled at the same time (Fig. 2a).

**Table 1:** Aerosol concentrations (particles.l^‐1^) for participants with cardiac arrest, participants under general anaesthesia, and pigs in a cardiac arrest model. Values are median (IQR [range]).

Event	Patients with cardiac arrest	Patients under general anaesthesia	Pigs in a model of cardiac arrest*
	n = 18	n = 16	n = 8
Breath one time‐average	5662 (1780–21,061 [814–87,189])	230 (100–683 [6–3395])	18,162 (2958–64,434 [65–95,204])
Breath two time‐average	7166 (4523–36,269 [541–149,535])	267 (104–575 [12–2459])	29,735 (2544–52,486 [1436–180,864])
Breath one peak	17,926 (5546–59,209 [1523–242,648])	744 (309–2106 [23–9099])	60,686 (5629–201,026 [114–364,049])
Breath two peak	16,151 (9335–81,598 [1215–321,662])	1106 (401–2222 [60–9164])	45,411 (7037–105,998 [2992–373,371])

Breath 1 is a single ventilatory breath and breath 2 also includes a period of chest compressions for participants with cardiac arrest and the pigs in a model of cardiac arrest.

* Aerosol concentrations recorded following cardiac arrest.

**Figure 3:**
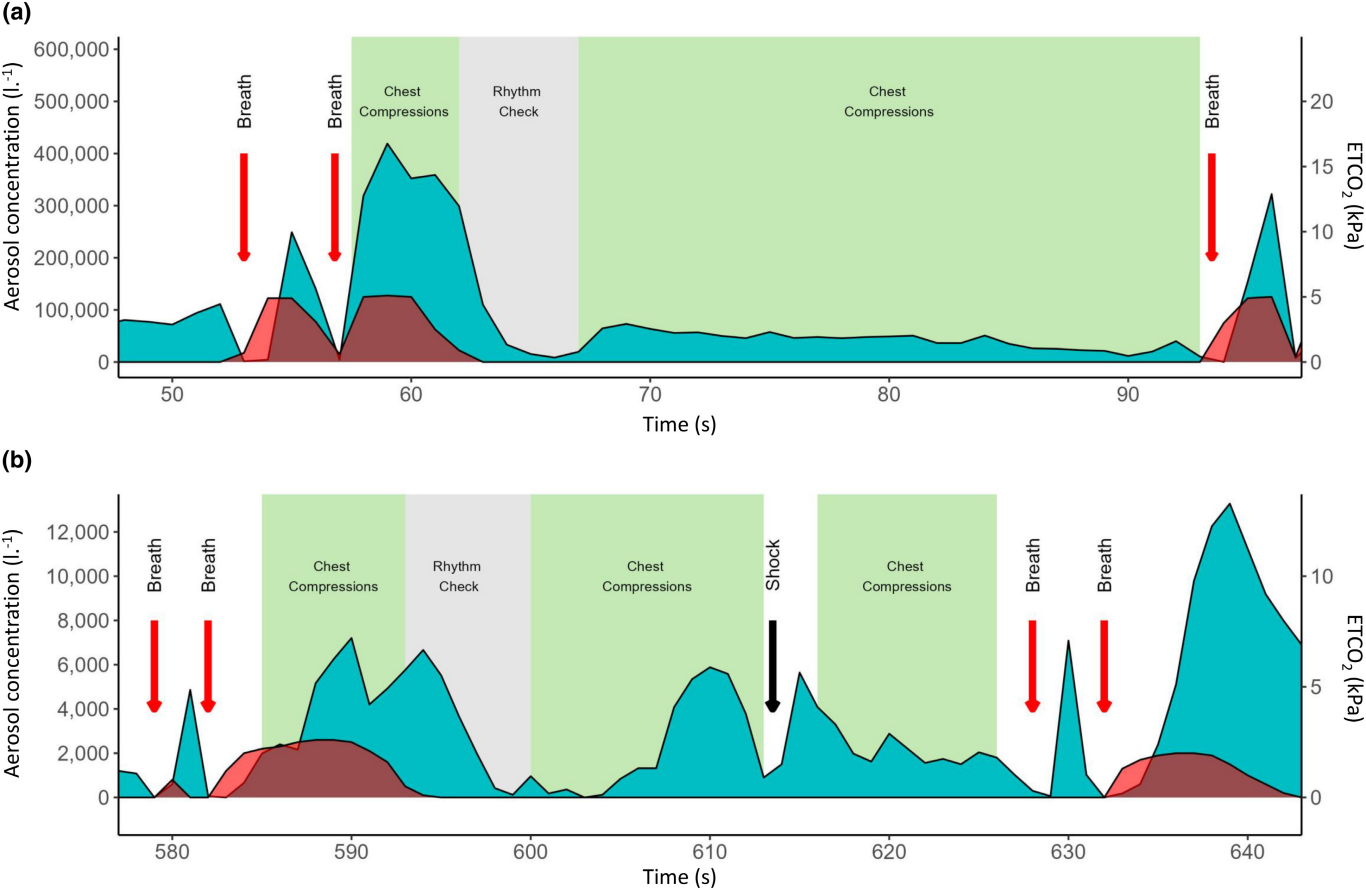
Timelines demonstrating aerosol concentrations and exhaled carbon dioxide values associated with chest compressions and defibrillation in human participants. Blue, aerosol concentration; red, ETCO_2_; red arrows, breaths. (a) Aerosol concentrations during a period of chest compressions following a 5 s rhythm check dissociating it from ventilatory breaths; (b) aerosol generation associated with external cardiac defibrillation during CPR (black arrow, external cardiac defibrillation shock). (Data shown in (a) and (b) are from two different participants in cardiac arrest).

Specific periods were identified during resuscitation when chest compressions were relatively isolated from ventilatory breaths, most commonly after a rhythm check (identified in 11/18 participants). When chest compressions resumed following the rhythm check (and in the absence of immediately preceding ventilatory breaths), there was a clear increase in the concentration of aerosol emitted, which was maintained above the baseline (11/11 recordings) (Fig. 3a).

Time‐average and peak aerosol concentrations during these periods of chest compressions were 1006 (503–2141 [60–77,511]) particles.l^‐1^ and 3798 (1158–7194 [180–142,903]) particles.l^‐1^, respectively. These values were lower than breath one in the same participants (time‐average 2764 (1644–5978 [814–87,189]) particles.l^‐1^, p = 0.002; and peak 9003 (5224–20,210 [1523–242,648]), p = 0.001).

Four participants with cardiac arrest had a single defibrillation shock (120–200 J) delivered during aerosol sampling. In all cases there was a transient increase in the aerosol concentration detected following the shock (Fig. 3b). Particle concentration analysis for the 10 s following the shock showed both time‐average (32,941 (9608–75,429 [2653–139,846]) particles.l^‐1^) and peak aerosol concentrations (72,128 (25,988–201,455 [5645–471,355]) particles.l^‐1^) were greater than those generated during breath one in the same participants with cardiac arrest (time‐average 13,871 (4896–25,652 [1603–37,361]) and peak 38,477 (16,913–68,633 [5237–106,084]) particles.l^‐1^); however, the small sample size (n = 4) precluded formal statistical analysis. It was also not possible to determine the effect of external cardiac defibrillation as an isolated event during resuscitation attempts because all shocks were preceded and immediately followed by chest compressions in accordance with resuscitation guidelines (Fig. 3b).

**Figure 4:**
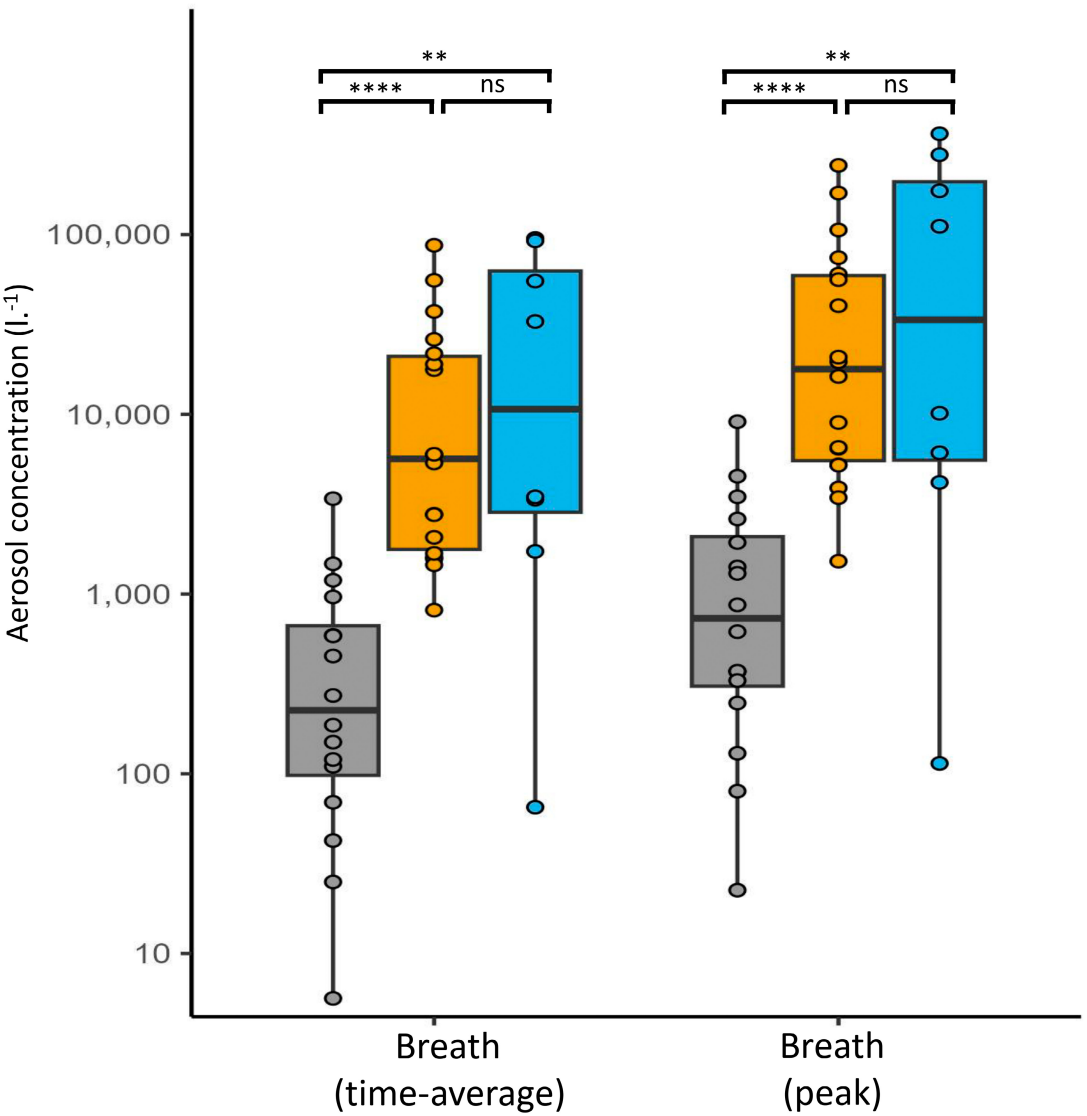
Average aerosol concentrations associated with isolated ventilatory breaths (breath 1 of the breath couplets). Bold line, median; boxplots show IQR; circles, individual data points; whiskers, range; grey, participants under general anaesthesia (n = 16); orange, participants in cardiac arrest (n = 18); blue, porcine cardiac arrest model (n = 8). ns = p > 0.05; **p < 0.01; ****p < 0.001.

Comparator aerosol data generated using an equivalent pattern of ventilation were collected in 16 anaesthetised patients (11 male, age 59 (52–66 [19–91]) y, BMI 28.1 (25.5–28.6 [17.6–39.0]) kg.m^2^). Time‐average and peak aerosol concentrations during ventilation of patients under general anaesthesia (breaths 1 and breaths 2) were more than an order of magnitude lower than those recorded in participants with cardiac arrest (all p < 0.001, Table 1 and Fig. 4).

Aerosol concentrations for breath one and breath two were not different in patients under general anaesthesia (Table 1), and the decay of breath two was more rapid and showed an almost identical profile to the decay of the ETCO_2_ waveform (Fig. 2b).

**Figure 5:**
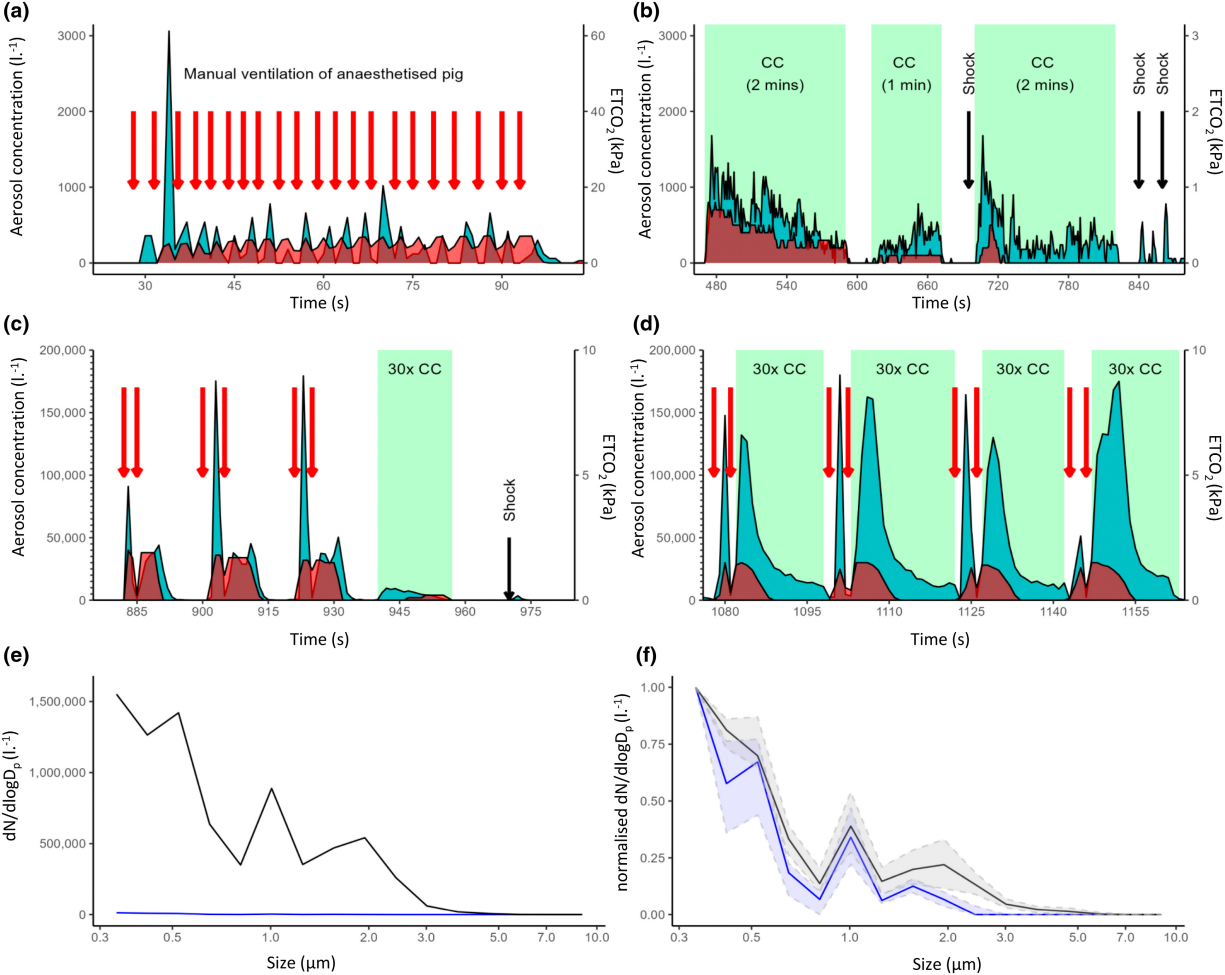
Representative respiratory aerosol concentration and ETCO_2_ during components of resuscitation in the porcine cardiac arrest model. Blue, aerosol concentration; red, ETCO_2_; red arrows, breaths; CC, chest compressions; shock, external cardiac defibrillation shock. (a) Aerosol recorded during ventilation of an anaesthetised pig before termination and starting CPR; (b) aerosol generation during: compression only CPR; compressions followed by external defibrillation; and isolated defibrillation shocks; (c) aerosol generated by sequences of two ventilatory breaths followed by a 20‐s pause (x 3) without any chest compressions. This is followed by 30 x chest compressions and a 20‐s pause; followed by an isolated defibrillation shock; (d) aerosol generated during cycles of two ventilatory breaths followed by 30 x chest compressions. Note the similar aerosol and ETCO_2_ profile to the human data in Fig. 2a; (e) comparison of the particle size distribution, during 60 s of ventilation of the anaesthetised animal vs. 60 s of ventilation following cardiac arrest. Aerosol concentrations normalised to dN/dlogD_p_ to account for the lognormal size distribution, n = 8. Blue line, pre‐arrest median; black line, post‐arrest median; (f) data in (e) normalised to the maximum value per animal. Blue line, normalised pre‐arrest median; black line, normalised post‐arrest median; shaded area, IQR. Note changes of y‐axis aerosol scale between (a–d) to best represent the data graphically.

Aerosol recordings were collected from eight Large White Cross female pigs during CPR (weight 83 (76–85 [53–95]) kg, age 5 (5–6 [4–6]) months). Time‐average aerosol concentrations during ventilation of the anaesthetised pigs just before euthanasia were 72 (41–136 [23–268]) particles.l^‐1^, values similar to those seen in participants under general anaesthesia (p = 0.070, Table 1, Fig. 5a). After cardiac arrest, isolated ventilation in pigs (without chest compressions) generated approximately 270‐fold higher aerosol concentrations than ventilation before cardiac arrest (19,410 (2307–41,017 [104–136,025]) particles.l^‐1^, p = 0.008). Peak and time‐average aerosol concentrations for the pigs following cardiac arrest were no different to those in human participants with cardiac arrest (p = 0.397 and p = 0.495, respectively) but higher than those seen in participants under general anaesthesia (p = 0.002 and p = 0.003, respectively) (Fig. 4).

Episodes of chest compressions in the porcine model (delivered before ventilation commenced) generated aerosol with a pattern and magnitude similar to that recorded during human CPR (time‐average 1584 (251–3084 [148–4459]) particles.l^‐1^, p = 0.967) (Fig. 5b). Chest compressions generated higher aerosol concentrations compared with ventilating the animal pre‐arrest, but lower than those produced by ventilation after cardiac arrest (breath 1) (p = 0.008 and p = 0.023, respectively).

External cardiac defibrillation of the pigs generated clear and reproducible transient spikes of aerosol time‐average concentration for 10 s following a shock (1677 (620–4041 [245–4576]) particles.l^‐1^) (Fig. 5b and c). Defibrillation generated higher aerosol concentrations than when ventilating the animal's lungs pre‐cardiac arrest but lower than post‐cardiac arrest ventilation (breath 1) (p = 0.008 and p = 0.039, respectively).

The phases of 30:2 CPR in pigs generated a repeating waveform of aerosol (and ETCO_2_) similar to that seen in patients with cardiac arrest (Fig. 5d). The addition of chest compressions to ventilatory breaths did not significantly change the peak aerosol concentrations detected 34,458 (5,738–108,105 [580–388,738]) particles.l^‐1^ without compressions vs. 45,411 (7037–105,998 [2992–373,371]) particles.l^‐1^ with compressions, p = 0.945). However, the addition of chest compressions prolonged the duration of aerosol emission, reflected in the time taken for the aerosol concentration to return to below 20% of the peak (mean (SD) 2.6 (0.8) s without compressions vs. 7.3 (3.0) s with compressions, p = 0.009).

Size distribution analysis of the particles generated during ventilation of the pigs showed an increase in all particle sizes following cardiac arrest; however, there was a marked increase in particles < 1μm (Fig. 5e and f). The profile of the particle distributions was similar between the pre‐arrest and post‐arrest recordings, providing further evidence they were respiratory in origin.

## Discussion

We found that CPR in humans generates high concentrations of respiratory aerosol and these concentrations were consistently higher than those seen in previous studies of awake and anaesthetised humans (by up to 100‐fold), even when coughing or undertaking forced expiratory activities [13, 14, 15, 16]. This finding is important because respiratory aerosols generated during CPR may pose a high risk of airborne infectious disease transmission. It should be noted that high aerosol concentrations do not necessarily mean high risk of airborne disease transmission and more work is required to determine the risk of airborne disease transmission from this aerosol and its environmental dispersion. However, as very high concentrations of respiratory aerosol are generated during CPR, we recommend airborne transmission precautions for providers in the setting of high‐risk pathogens, until the airway is secured and sealed with an advanced airway device and breathing system with a filter.

We extended our observational findings in human patients to a porcine cardiac arrest model. This model facilitated paired respiratory aerosol measurements before and after cardiac arrest (recordings not possible for participants recruited following cardiac arrest). Here, we showed that ventilatory breaths delivered after cardiac arrest generate 270‐fold higher aerosol concentrations than those before cardiac arrest, strongly suggesting it is the physiological changes following cardiac arrest that cause the high aerosol concentrations measured. As these recordings were undertaken within the same animal they account for inter‐subject variability, increasing the confidence in the findings. The porcine model also enabled each component of CPR to be studied both in isolation and as part of the standard CPR algorithm directly analogous to that delivered to human participants; this is not possible during CPR for patients with cardiac arrest because it would be a deviation from standard care that could compromise outcomes. These porcine recordings show isolated chest compressions and external cardiac defibrillation generate aerosol but at lower concentrations than during the ventilatory components of CPR. There was, however, a synergistic interaction when ventilatory breaths, chest compressions and external defibrillation are combined (as also reported in a recent porcine CPR model [28]).

Delivering manual chest compressions requires significant physical activity; rescuers are close to the patient and likely to be taking large volume breaths increasing the risk of inhaling aerosol generated during CPR. It is likely that chest compressions, with or without external cardiac defibrillation, will release aerosol near the rescuer at the start of resuscitation before the airway is secured. Furthermore, the delivery of ‘rescue breaths’ without a filter, whether by mouth‐to‐mouth or mask ventilation, are likely to be associated with a particularly high‐risk of aerosol inhalation and potential for acquisition of respiratory pathogens.

Our previous studies of respiratory aerosol emissions were undertaken in ultraclean operating theatres, an environment with very low (practically zero) background aerosol concentrations, enabling resolution of aerosol generated by natural respiratory events such as breathing, speaking and coughing [13, 14, 15, 16, 23, 24, 29]. These studies show the presence of detectable respiratory aerosol at distances of 20 cm associated with tidal breathing and 50 cm with coughs in healthy subjects (who generate far lower aerosol concentrations than those detected during cardiac arrest) [13, 14, 15, 16, 24]. The inference is that during CPR, bioaerosol will be detectable in much larger quantities and so poses an appreciably higher risk of aerosolised disease transmission; however, once an airway device with a filter is inserted, it is likely that the aerosol will be contained. Nonetheless, caution when breaking the breathing circuit (e.g. when disconnecting the oxygen for defibrillation) is likely to be prudent, unless the filter is left attached to the airway device.

We suggest that the high aerosol concentrations detected during ventilation in cardiac arrest are due to re‐expansion of collapsed airways, generating respiratory aerosol via the bronchiolar fluid film burst mode [30, 31]. As collapsed alveoli re‐expand, physiological fluid (surfactant and oedema) ‘bursts’ into small particles which are expelled with the next exhalation. The loss of muscle tone in the period before and during cardiac arrest, administration of 100% supplemental oxygen and external chest compressions, all promote distal airway collapse, an early event in cardiac arrest which impairs ventilatory gas exchange [32, 33]. Subsequent cycles of lung re‐expansion from manual ventilation will increase aerosol generation via the bronchiolar fluid film burst mode; the increase in submicron aerosol particles seen during ventilation of the pigs following cardiac arrest supports this theory.

Additionally, the application of external chest compressions rapidly expels turbulent air at high velocity from within the lung resulting in respiratory aerosol emission, and external cardiac defibrillation produces strong diaphragmatic contraction mimicking a short, sharp inhalation–exhalation with an associated release of respiratory aerosol. This pathophysiology is an inevitable consequence of cardiac arrest and the components of resuscitation. The application of positive end expiratory pressure may reduce aerosol emissions by splinting open lung units but is unlikely to eliminate their generation and would require further research.

Background environmental aerosol concentrations are typically much higher than those generated by respiratory events, fluctuate greatly over time and vary considerably at different locations [29, 34]. The use of ultraclean environments in our previous studies eliminated the effect of background aerosol. It was necessary, however, to adapt our methodology as cardiac arrest is both an unpredictable event and rare in operating theatres. Using a novel sampling setup (validated by our study group in awake participants) we were able to eliminate the influence of background aerosol by sampling directly from the respiratory circuit [23]. The inline bacterial and viral filter removes particles > 0.3 μm (the lower limit of detection of the aerosol sampling device) from the inspired gas, giving confidence that all aerosol detected was participant‐generated.

It should be noted that our study has only measured respiratory aerosol and not assayed the presence of transmissible pathogens such that we can only make inferences about transmission risk. However, patients with an acute viral illness generate more respiratory aerosol, and respiratory pathogens such as SARS‐CoV‐2 are concentrated in the smaller aerosol particles, generated predominantly in the distal respiratory tract by the bronchiolar fluid burst mechanism [35, 36]. All aerosol detected in this study was in the respirable size range (< 10 μm diameter) and likely to pose a substantial risk to first responders from those patients with cardiac arrest who have a transmissible respiratory pathogen.

Our sampling methodology meant that assessments could not be made during the early bystander or ‘first responder’ phase of CPR. However, sampling air from the vicinity of the participant in the natural environment would present major challenges to ascertain whether detected aerosol originated from their respiratory tract. Advances in aerosol sampling technology (such as biofluorescence signatures) may enable future assays to distinguish respiratory aerosol from other particles, but this is not yet possible. It is likely that the degree of aerosol emission will be affected by airway patency when CPR is first started (with a similar effect on gas exchange) but this should not be considered a sufficient mitigation for risk, as it is likely to be unpredictable from case to case. It should be noted that CPR aerosol dispersion models and simulation studies require prior knowledge of the amount of aerosol emitted at the source; our study has, to our knowledge, provided these data for the first time.

Our sample sizes are relatively small, reflecting the challenges in collecting these unique data but are in line with typical sample sizes in other clinical aerosol studies. Furthermore, the large differences detected in aerosol generation with specific components of CPR increases confidence of our findings. The observational human cardiac arrest data were supported by the porcine model to enable separation of the various CPR components. The animals included in the study were of a similar weight to the participants under general anaesthesia; respiratory aerosol concentrations generated by ventilation of the pigs before cardiac arrest were similar to those seen during ventilation of human participants under general anaesthesia; respiratory aerosol concentrations generated during CPR manoeuvres in the porcine cardiac arrest model were similar to those measured during resuscitation of participants with cardiac arrest; and the profile of aerosol detected during porcine CPR replicated that seen in human participants.

All of the participants with cardiac arrest had bystander CPR and a prolonged period before aerosol sampling (typically around 30 min), which is likely to have resulted in substantial lower airway collapse by the time the recordings were made (and is likely associated with the poor outcomes observed in this group) [33]. However, the high aerosol concentrations detected in the porcine model were similarly elevated (recorded within 2–3 min of arrest), indicating that the key pathophysiological changes manifest quickly as might be expected for alveolar collapse.

We have shown previously that airway management manoeuvres, including facemask ventilation, insertion of airway devices or airway suctioning, are low‐risk for aerosol generation and should not be classified as aerosol‐generating procedures [13, 14, 15, 16, 18, 19]. In contrast, we have identified that very high aerosol concentrations are generated during resuscitation of participants with cardiac arrest. Our study indicates that is the re‐expansion of collapsed airways associated with the pathophysiology of cardiac arrest that leads to aerosol generation, and not the airway manoeuvres. We have summarised our findings and recommendations in Box 1.

Box 1 **Findings and recommendations.**

- Cardiopulmonary resuscitation generates very high concentrations of respiratory aerosol.
- These high aerosol concentrations potentially increase the risk of airborne disease transmission.
- We recommend airborne transmission precautions during CPR in the setting of high‐risk pathogens.
- Risk is reduced once the airway is secured and connected to a breathing system with a filter.

## Supporting information


**Appendix S1.** Porcine cardiac arrest CPR protocol.
