# Supplementary material for: A quantitative evaluation of aerosol generation during cardiopulmonary resuscitation
Source: Anaesthesia. 2023 Nov 3;79(2):156–67. doi: 10.1111/anae.16162 (PMC10952244; doi:10.1111/anae.16162)
Supplement: Supplementary file 1 — Appendix S1. Porcine cardiac arrest CPR protocol. [file ANAE-79-156-s001.docx]

**Appendix S1** – Porcine cardiac arrest CPR protocol

| **Event** | **Duration (seconds)** |
| --- | --- |
| Manual ventilation with aerosol sampling device | 60 |
| Disconnect Sampling device |  |
| Euthanasia as per project license with Sodium Pentobarbital. |  |
| Confirm arrest then administer 50mmol KCl with saline flush |  |
| External chest compressions | 120 |
| Pause | 20 |
| External chest compressions | 60 |
| Pause | 20 |
| External cardiac defibrillation | ~5 |
| External chest compressions | 120 |
| Pause | 20 |
| External cardiac defibrillation | ~5 |
| Pause | 20 |
| External cardiac defibrillation | ~5 |
| Pause | 20 |
| 2 breaths | ~10 |
| Pause | 20 |
| 2 breaths | ~10 |
| Pause | 20 |
| 2 breaths | ~10 |
| Pause | 20 |
| 30x compressions | ~20 |
| Pause | 20 |
| External cardiac defibrillation | ~5 |
| Pause | 20 |
| 2 breaths then 30 compressions (x2) | ~60 |
| Pause | 10 |
| 30 x compressions | ~20 |
| External cardiac defibrillation | ~5 |
| 30 x compressions | ~20 |
| 2 breaths then 30 compressions (x4) | ~240 |
| Pause | 10 |
| External cardiac defibrillation | ~5 |
| Pause | 20 |
| **Total protocol sampling time** | 17mins (approx.) |
